# Glanders in Horses

**Published:** 1896-04

**Authors:** E. H. Biart

**Affiliations:** Lansing, Kansas


### GLANDERS IN HORSES.[1]

BY DR. E. H. BIART,
LANSING, KANSAS.

WHILE the contagious nature of glanders has been admitted from the earliest period, it seems from the recent observations and experience of the veterinary surgeon in charge of the Veterinary School and Hospital at Lyons that glanders is not as highly contagious or infectious as it has been supposed to be, for of a hundred horses exposed to the contagion of the disease only eight contracted the affection. But all medical men will admit that the propagation of any contagious disease in a great measure depends upon the predisposing causes that may exist at the time. It is probable that the horses exposed to the contagion were in robust health, possessing in a large degree that vital power which enables them to resist contagion. The principle of contagion in glanders, whether strong or weak, is admitted by all observers; and we believe it is generally the exciting cause. Nevertheless, as a general law, it may be stated that as is the case with smallpox and other contagious diseases incident to man, so it is with the first horse that suffers with glanders: it must necessarily have taken it without contagion. So it may occasionally be engendered by causes of which we are ignorant.

It may be stated, also, that contagion, once generated, multiplies itself without any assignable limit. It may be proper to explain what is meant by contagion. It is here used in its specific sense, meaning the direct transmission of disease by means of a specific virus from one animal to another by natural or artificial inoculation. Horses recognize and communicate with each other by the sense of smell. It is in this way, perhaps, that the mucous membrane of the nose becomes the first apparent seat of glanders. The glandered horse blows the virus from his nose on everything around him, and the disposition of these animals to recognize animal matter wherever found induces the sound horse to investigate the character of the noxious secretions. He does this by a strong inhalation; the virus is in this way drawn up and retained upon the delicate mucous membrane of the nose, or is, perhaps, caught by the

[1] Read at the Missouri Valley Veterinary Association's meeting.

roughened surface of an abrasion; it finds its proper nidus and multiplies its morbific influence. It is possible that a horse may have glanders for several weeks before it becomes an agent of active contagion, from the fact that the disease is often slow and insidious in its onset; and it is only when the mucous membrane of the nose becomes irritated by chancrous ulcers that the snorting out of the virulent matter occurs, every surrounding object then becoming a deposit for the virus.

The disease commences very insidiously, but increases steadily in virulent malignancy to rapid decomposition of the blood, exhaustion, and death. The first symptom that attracts attention to an animal in the onset of an attack of glanders is a watery discharge from the nostril, without fever, loss of appetite, or unusual disturbance of any kind. In every instance of my observation coincident with the watery discharge of the nostril I have found the lymphatic glands beneath the angle of the lower jaw on the side corresponding to the affected nostril engorged, with a tendency to become adherent to the adjacent branch of the jaw-bone; they may be movable up and down, but confined and restricted by the adhesions to very limited movements, and they never suppurate or form abscesses. In this stage of the disease the mucous membrane of the nostrils will have lost its natural color, and most frequently, but not invariably, presents a pale-buff aspect with purple dots or patches of congestion as it extends up the cavity of the nose, and these patches, on close examination, will be found to be an aggregation of small overdistended bloodvessels. In many instances the mucous follicles will seem elevated on the surface of the membrane of the nose, and sometimes will present a purple hue.

Several days may elapse, the animal still without fever, cough, or loss of appetite; the coat may stare or become rough, and there is appearance of ill-health. The watery discharge of the nostrils becomes more abundant and glutinous, the lymphatics become more engorged and adherent to the bone; the congestion of the nasal mucous membrane increases; the most prominent follicles degenerate into small ulcers; and the discharge soon assumes a thick, yellow, purulent character and becomes still more abundant. Cough now supervenes, more or less frequent, produced by the general irritation and inflammation of the air-passages, and the tendency to sneeze and snort out the purulent matter begins.

The disease may now progress with this continuous purulent discharge from the nose for weeks, and indeed for months, while the ulceration is extending throughout the course of the mucous membrane into the remotest cavities of the nose, in the interior portion of the skull. During this time hemorrhage of the nose often occurs and proves very exhausting to the horse. The constitution is now gravely affected, the animal becomes emaciated, the legs swell, and swellings occur about the head and lips. Generally by this time the blood becomes so poisoned that death soon occurs.

Of the 100 horses exposed to the disease, as before mentioned, only 8 caught it by infection, and at another trial where an almost equal number were exposed, as experiments, 20 of the exposed animals were affected. But in every instance where horses were inoculated artificially or exposed to direct contagion they invariably caught the disease. This goes to prove beyond a doubt that glanders in horses is far more contagious than infectious.

